# A mathematical model by route of transmission and fibrosis progression to estimate undiagnosed individuals with HCV in different Italian regions

**DOI:** 10.1186/s12879-022-07042-w

**Published:** 2022-01-17

**Authors:** Loreta A. Kondili, Massimo Andreoni, Alfredo Alberti, Salvatore Lobello, Sergio Babudieri, Antonella De Michina, Rocco Merolla, Walter Marrocco, Antonio Craxì

**Affiliations:** 1 National Center for Global Health, Istituto Superiore di Sanità, Rome, Italy; 2 University of Tor Vergata, Rome, Italy; 3 Department of Molecular Medicine DMM, University of Padova, Padua, Italy; 4 Health Unit Euganea, Padua, Italy; 5 University of Sassari, Sassari, Italy; 6 Medical Department AbbVie Italy, Rome, Italy; 7 F.I.M.M.G., Rome, Italy; 8 Gastroenterology and Liver Unit, DiBiMIS, University of Palermo, Palermo, Italy

**Keywords:** HCV, Undiagnosed, Hepatitis C infection, Prevalence, Markov chain

## Abstract

**Background:**

Although an increase in hepatitis C virus (HCV) prevalence from Northern to Southern Italy has been reported, the burden of asymptomatic individuals in different Italian regions is currently unknown.

**Methods:**

A probabilistic approach, including a Markov chain for liver disease progression, was applied to estimate current HCV viraemic burden. The model defined prevalence by geographic area using an estimated annual historical HCV incidence by age, treatment rate, and migration rate from the Italian National database. Viraemic infection by age group was estimated for each region by main HCV transmission routes of individuals for stage F0–F3 (i.e. patients without liver cirrhosis and thus potentially asymptomatic) and F4 (patients with liver cirrhosis, thus potentially symptomatic).

**Results:**

By January 2020, it was estimated that there were 409,184 Italian individuals with HCV (prevalence of 0.68%; 95% CI: 0.54–0.82%), of which 300,171 (0.50%; 95% CI: 0.4–0.6%) were stage F0–F3. Considering all individuals with HCV in stage F0–F3, the geographical distributions (expressed as the proportion of HCV infected individuals by macroarea within the overall estimated number of F0–F3 individuals and prevalence values, expressed as the percentage of individuals with HCV versus the overall number of individuals for each macroarea) were as follows: North 42.1% (0.45%; 95% CI: 0.36–0.55%), Central 24.1% (0.61%; 95% CI: 0.48–0.74%), South 23.2% (0.50%; 95% CI: 0.4–0.61%), and the Isles 10.6% (0.49%; 95% CI: 0.39–0.59%). The population of people who inject drugs accounted for 50.4% of all individuals infected (F0–F3). Undiagnosed individuals (F0–F3) were ~ 15 years younger (⁓ 50 years) compared with patients with stage F4 (⁓ 65 years), with similar age distributions across macroareas. In contrast to what has been reported on HCV epidemiology in Italy, an increasing trend in the proportion of potentially undiagnosed individuals with HCV (absolute number within the F0–F3) from South (23.2%) to North (42.1%) emerged, independent of similar regional prevalence values.

**Conclusion:**

This targeted approach, which addresses the specific profile of undiagnosed individuals, is helpful in planning effective elimination strategies by region in Italy and could be a useful methodology for other countries in implementing their elimination plans.

**Supplementary Information:**

The online version contains supplementary material available at 10.1186/s12879-022-07042-w.

## Background

Hepatitis C virus (HCV) is the leading cause of liver-related morbidity and mortality worldwide. Globally, as many as 71 million individuals are infected with chronic HCV [1, 2], Many HCV infected individuals remain asymptomatic for decades, although progression of the disease accelerates with age [3, 4].

Following the availability of direct-acting antiviral drugs (DAAs) for the successful treatment of HCV infection [5, 6], focus has been placed on the identification of infected individuals. The identification of this population is necessary to achieve targets for 2030, established by the World Health Organization [7].

Italy is considered the country with the highest HCV prevalence rate in Western Europe [8–10] and we have previously published national estimates on the number of infected individuals using a similar modelling approach [11].

It has previously been estimated that under an assumption of 40% of infected individuals linked‐to‐care, viraemic burden would decline by 60% and eligible patients to treat will be depleted around 2025 leaving a significant proportion of infected individuals undiagnosed and without care [12]. To achieve HCV elimination goals, increased case finding in targeted, high prevalence groups is required.

In Italy, although a national hepatitis plan exists [13], decentralized models of HCV are still being implemented without any uniform screening strategies exist across regional networks. There is a dedicated fund for free HCV screening approved by law in Italy which needs to be implemented at the Regional level and to this end an estimate of the number of individuals with HCV for each region is necessary. To address this unmet need, we used a probabilistic approach to estimate the infection rate and a Markov model for liver disease progression. This mathematical modelling approach can be considered a useful tool to aid in the development of national and regional health authority HCV elimination plans.

## Methods

### Study design

A previously validated and published mathematical modelling has been used for the aims of this study [11]. Specifically, the mathematical model employed in the present study may be split into two distinct computations. The first model (previously described elsewhere [11]) computes the number of infected individuals on a national basis from available literature and Italian National database [14], subdivided per age, route of transmission, fibrosis status from 1952 to October 2019. Data from the Italian National Institute of Statistics database ISTAT [14] on internal migration from one region to another were only available from 2002 onwards. Therefore, based on the estimation of the number of infected individuals on a national basis until year 2001, the regional evolution of HCV transmission and progression (including inter-regional and international migration) starts from the year 2002. The second model, the results of which are presented in this paper, compute the results as previously described [11] until the year 2001 and from the year 2002 also provide the number of infected individuals on a regional basis, subdivided by age, route of transmission, fibrosis status, up to January 2020.

### Study population and literature search

Data on HCV prevalence for routes of infection in high risk groups was obtained from a literature search previously reported in detail [11].

### The model

**Fig. 1:**
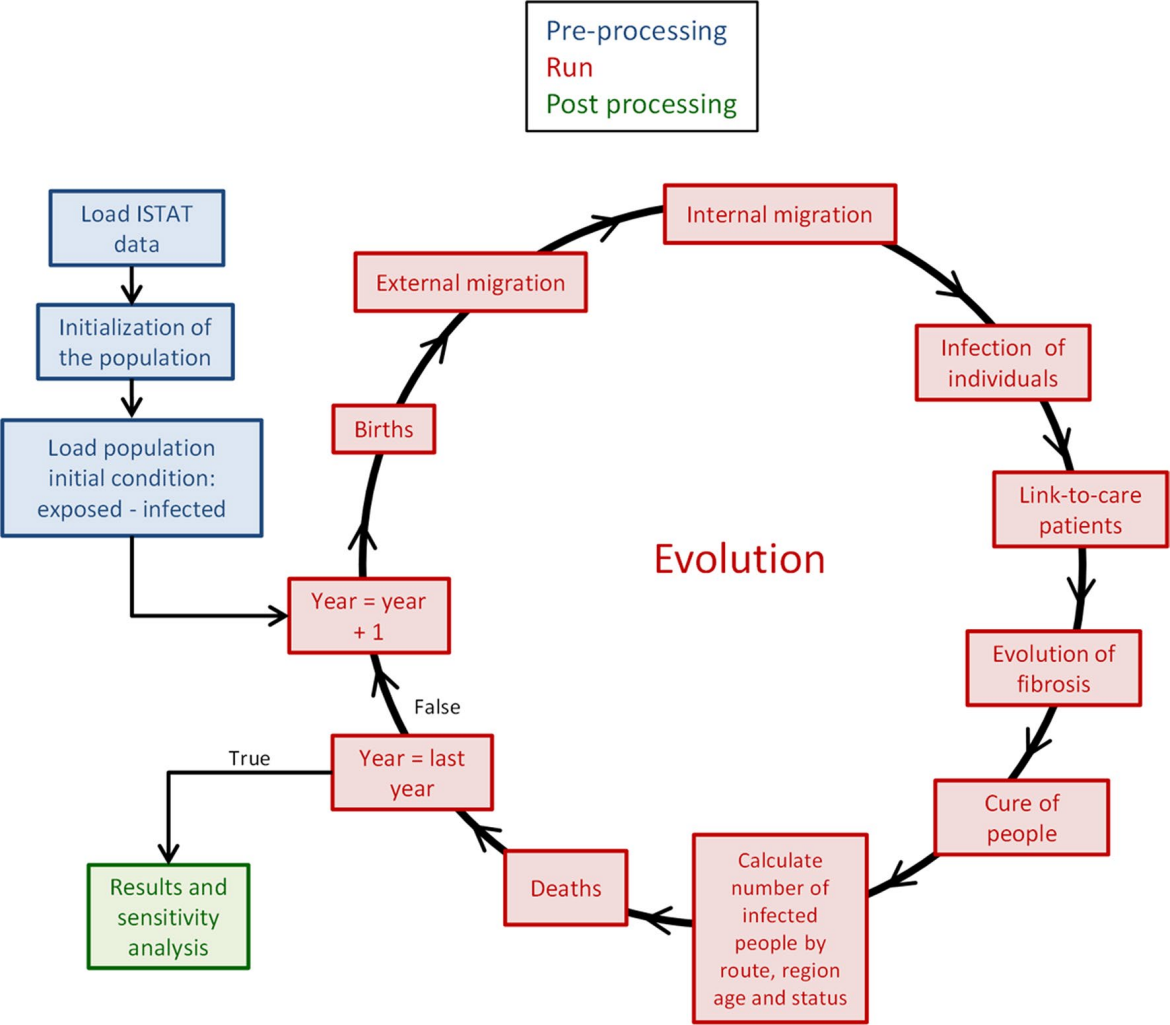
Schematic presentation of the model evolution loop

An evolutionary HCV transmission model was developed and implemented using the open-source programming language Python 3.7. The first stage of the model (termed *national phase* here forward) has been previously reported [11], beginning in 1952 to the end of 2001 calculating the number of infected individuals by age, route of transmission, and fibrosis status. Briefly, the evolutionary steps consider the insertion of newborns, new infections (the model accounts for six routes of transmission), possible fibrosis evolution (computed using a Markov chain approach), HCV treatment, and liver-related mortality rate. After this stage, the number of individuals with HCV is subdivided for each region considering the distribution of the population and the variability of risk factors among the different regions is explained in section “Transmission routes and associated risk”. Subsequently, the evolution of the HCV transmission and liver disease progression among the Italian population was performed until January 2020, with a method that included the internal (inter-regional) and external (international) migration referred to as regional phase here forward which consists of several steps summarised below with a graphical representation depicted in Fig. 1.

The evolutionary steps performed by the national and regional phases are nearly the same. The regional phase is more accurate and computationally intensive because it includes migration flow.

An overview of these steps is described below.

1. Newborns: The number of newborns is added, official annual data are provided by ISTAT [14].
2. External migration (only for the regional phase): the movement of people from a foreign country to Italy or vice versa. People who enter Italy are considered healthy; however, they can contract HCV once they arrive, just as other Italian residents. People who leave Italy are simply removed from the model; they either have or do not have HCV. ISTAT provides a minimum breakdown by age, which is included in our model [14].
3. Internal migration (only for the regional phase): the movement of people from one Italian region to another. People who move between regions can contract HCV. Because ISTAT [14] does not provide information on internal migrants’ ages, we pick them independently of their age.
4. Infected individuals: individuals recently contracting HCV per region, age, and year were considered [11, 15]. These individuals are added to the previous population of individuals with HCV. The number of new HCV cases was then calculated following the infection probabilities, depending on the age, transmission route, and current year.
5. Link-to-care patients: the burden of individuals with HCV treated with interferon (IFN) or DAA provided by Expert Opinion report provided by EpaC (The Italian Liver Patients’ Association [EpaC]) and the Italian Medicines Agency (AIFA) Registry for DAA monitoring [16].
6. Fibrosis evolution: individuals with HCV who undergo a possible fibrosis progression according to a Markov chain probability evolution process [11].
7. Mortality: the annual number of deaths per region and age provided by ISTAT [14] was removed. Individuals were randomly (uniform) selected between the populations of those with and those not with HCV. In our model, we accounted for HCV-related mortality by considering the average values of transition probabilities following the F4 fibrosis stage reported by Linthicum et al. [17] and Kondili et al. [18–21]. All transition probabilities were adjusted for competing probabilities of death from other causes according to the official data [ISTAT] [14].
8. Year + 1: individuals are aged 1 year.

Subsequently, the ‘loop’ restarts, advancing the population for another year. This process repeats until the year 2020 (Fig. 1).

### Transmission routes and associated risk

The model tracked each single route of transmission independently, distributing the weight of the effect of each route over time, as previously described in detail elsewhere [11]. The probability, *P*, of contracting *HCV* after exposure to a route, in a given year for a person of a given age, can be split as follows:

$$P\left(HCV|route,age,year\right)=g\left(HCV|route,age\right) \times f\left(HCV|route,year\right),$$

where both *g* and *f* are shape functions that express the dependence of *P* on the couples: “route – age” and on “route – year of infection”. The functions *g* and *f* have been described (and plotted) in further detail elsewhere [11].

Data for the following high-risk routes of HCV transmission were considered: people who inject drugs (PWID), tattoos or body piercing, sexual transmission, glass syringe, blood transfusion, and vertical transmission [11]. Key criteria for input of data in the model regarding age and year of infection were defined for each risk group. Based on HCV prevalence and the time series of Italian population, we reconstructed the probability of infection for ages 0–100, for years from 1952 to 2020, and for the six different infection routes. The formula used follows:

$$Newly\, infected\left(route, year\right)= \sum_{age}\left[g\left(HCV|route,age\right) \times f\left(HCV|route,year\right)\times F\left(route\right)\times I\left(route\right)\right],$$

where *F* and *I* are the fraction of the total Italian population exposed to the route and the viraemic population, respectively. For the regional phase, the number of newly infected individuals is distributed among regions proportionally to the population for each age and each region. The fractions of individuals exposed, the viraemic populations, as well as the functions *f* and *g*, are obtained from assumptions based on previous studies and described in detail elsewhere [11].

The total burden of infection at the beginning of 2002 was obtained using the results of the national phase [11], divided for each region. The formulas and criteria used to distribute the burden of infection across different regions are described below for each high-risk route of infection.

#### People who inject drugs

Regional distribution of PWID is estimated according to the 2002 national report on drug addiction (‘*Relazione Annuale Al Parlamento Sullo Stato Delle Tossicodipendenze in Italia*’) [22]. This report documents the number of people per region undergoing treatment for a drug addiction together with HCV. We distributed the number of infected individuals proportionally to the number of individuals under treatment in a SERD (‘*Servizi per le Tossicodipendenze*), both positive and not positive, in the formula:

$$PWID\left(region\right)={PWID}_{national}\times \frac{SerT(region)}{\sum_{all\, regions}SerT(region)}.$$

SERD (region) is the number of individuals undergoing treatment in a regional SERD, PWID (region) is the number of PWID individuals per region, and PWID_national_ is the overall number of PWID from the national phase. Therefore, we assume that the number of individuals receiving treatment for substance use disorders at a given centre for a specific region is directly proportional to the total number of PWID in that region. Furthermore, in the computation of the number of infected PWIDs, the number of those treated over time (since the use of IFN then of DAA) were eliminated from the computation.

#### Tattoo or body piercing

Regarding the regional phase, data were not available to evaluate the prevalence of this route of infection across different regions. Hence, the number of infected individuals were subdivided proportionally to avoid erroneous estimates. A region yields the number of those infected for a given age group proportionally to the number of those of that age in the region in the following formula:

$${N}_{infected}\left(region,age\right)=\frac{people(region,age)}{{\sum }_{all\, regions}people(region,age)} {N}_{infected-national}\left(age\right).$$

The relative importance of this route for the new infections is decreasing rapidly (in 2019 it is ~ 1/5 of the peak value reached in 1995) [11]. However, we cannot refer to this route as “extinct”, because it is dependent upon hygiene conditions of individuals performing tattoos and body piercing.

#### Sexual transmission

For the regional phase, we adopted the same formula used for the tattoo and body piercing route because, as with that route, data were not available to distribute the prevalence of the sexual transmission route of infection across different regions [11].

#### Glass syringes

A higher prevalence was assumed to have occurred at an early age (0–8 years) due to glass syringe vaccination. Ten percent of individuals were assumed to be exposed to glass syringe and 6% to be infected [11]. Because infection by glass syringe (in 1975 single use of plastic disposable syringes became law in Italy, substituting glass syringes nationwide) or blood transfusion routes did not result in any new HCV cases from 2002, these routes can be referred to as ‘extinct’ for the regional phase. Considering a recent study by Andriulli et al. [23] where a North–South gradient was observed (higher in the South) with a ratio of South: North of 1.14 and applying this information to our model, a ratio of 1.17 is generated. This value was obtained by ascribing a prevalence value three-fold higher for Southern regions compared with Northern regions.

#### Blood transfusion

The distribution profile is the same as that used previously [11]. The year distribution profile starts when the model begins and it peaks in the nineties (when the virus was finally isolated) and it drops smoothly in subsequent years. The regional distribution of the risk has been assumed to be uniform.

#### Vertical transmission

The risk of vertical transmission was calculated from the number of mothers with HCV; this female population transfer the virus with a given percentage to newborns (values retrieved from ISTAT]). The risk was estimated to be around 5.8% [14, 24]. Reduction of the risk has been modelled with a linear decrease up to 0.015% [11]. It is also reasonable to assume that the number of infected individuals will be proportional to the specific population for each region. Vertical infection occurs at the age of zero, but the infected population undergoes ageing, hence the risk of vertical transmission occupies any age bins.

### Patients treated with anti-viral drugs

Among the estimated number patients treated with anti-viral therapy, IFN- and DAA-based treatments were subtracted from the population with HCV, since they are not considered as an ‘unknown’ population anymore. For some of the years considered, literature and available data sources [11] gives an indication of how this number is distributed among the different fibrosis stage groups; otherwise we used a uniform distribution. From the estimated total number of viraemic patients at risk, we subtracted the number of probable non-viraemic individuals from the model (IFN- and DAA-based treatment) from 1993 to 2019 according to expert report data for IFN treatment until 2015 by EpaC (The Italian Liver Patients’ Association [EpaC] [25]) and AIFA Registry data for DAA monitoring [16]. For the period spanning from 2002 to 2014, since we rely on expert opinion, regional treatments have been calculated proportional to the number of infected individuals for each region and they have been uniformly distributed among the 5 fibrosis stages. Starting from 2015, we rely on data from AIFA [16] and regional treatments previously presented (ACE conference 4-November-2019) [26]. Treatments are uniformly distributed between the different routes.

### Markov chain progression for fibrosis evolution

The evolution from one stage to the next is modelled according to a Markov chain approach as has previously been described in detail [11]. Briefly, the following annual transition probabilities have been used in the reference case: F0 → F1 = 7.6%, F1 → F2 = 9.5%, F2 → F3 = 10.8%, and F3 → F4 = 13.4% [17]. It was assumed that no more than one transition per year could occur per patient. Potential spontaneous liver fibrosis regression was not considered.

### Sensitivity analysis

Sensitivity analysis was performed using a Monte Carlo approach to estimate the effect on the number of individuals with HCV and their annual distribution among the F0-F4 stages, the uncertainty in the fibrosis annual transition probabilities, and the probability of self-curing (recover spontaneously without treatment). Two thousand simulations were run. In each simulation, the self-curing and transition probabilities were derived (independently) from their predefined random distributions.

The distributions of the transition probabilities were logit-normal with mean value equal to the corresponding transition probability used in the reference case [17] and standard deviation equal to 10% of the mean value. The distribution of the self-curing probability was uniform, with lower bound at 15% and upper bound at 45% (thus, the mean value was 30%, as in the reference case [17]). The output of the Monte Carlo consisted of mean values of the different prevalence values computed using our model, together with a 95% confidence interval (CI), from 2.5th to 97.5th percentiles for each one of them.

## Results

### HCV prevalence estimates by Italian region and macroarea

The overall number of individuals with HCV by January 2020 in the entire Italian territory, estimated at 409,184 (prevalence of 0.68%; 95% CI: 0.54–0.82%), was distributed across the four macroareas as follows: 0.47% to 0.67% in the North, 0.74% to 1.04% in the Central, 0.64% to 1.01% in the South, and 0.66% to 0.76% in the Isles regions. Highest prevalence estimates (percentage of infected individuals versus the overall number of individual inhabitants in a given region) were generally observed in Central Italy, such as Umbria (1.1%, 95% CI: 0.88–1.34%) and Marche (1.04%, 95% CI: 0.83–1.26%), in addition to the Basilicata region in the South (1.01%, 95% CI: 0.81–1.22%), whereas the majority of Northern regions had a lower estimated prevalence (e.g. Lombardia; 0.47%, 95% CI: 0.38–0.57% and Emilia Romagna; 0.51%, 95% CI: 0.41–062%).

**Fig. 2:**
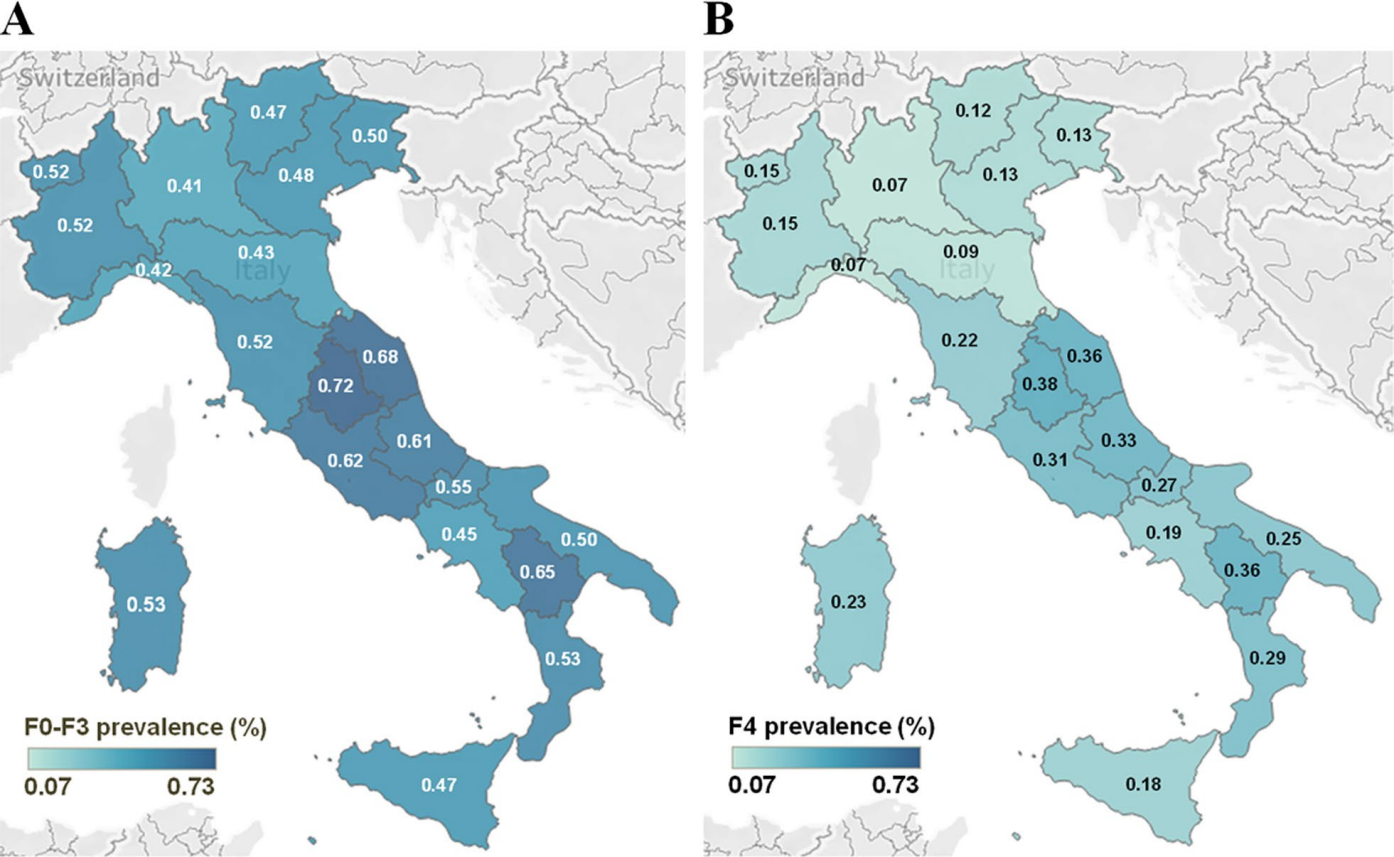
Maps of the estimated prevalence of HCV in different regions of Italy. **A** The prevalence of potentially undiagnosed individuals with HCV (stage F0–F3) and **B** individuals with HCV (stage F4 liver fibrosis; potentially diagnosed) are depicted in the two maps. Prevalence estimates were calculated according to each of the region’s population by age group annually since 1952 up to January 1, 2020, from the Italian National database (ISTAT) [14]. F0–F3 = asymptomatic; undiagnosed/unlinked to care; F4 = symptomatic; potentially linked to care and cure; HCV = hepatitis C virus. Please note that when referring to prevalence values for a given area (e.g. a region or a macroarea) we refer to the percentage of individuals located in that macro area that are infected

The distributions of HCV prevalence in individuals potentially asymptomatic (stage F0–F3, i.e. patients without liver cirrhosis; estimated to be 300,171) and those with F4 stage disease (and F4 (patients with liver cirrhosis, thus potentially symptomatic; estimated to be 109,012) are shown in Fig. 2A, B, respectively.

The overall HCV prevalence estimates (percentage of individuals with HCV versus the overall number of individuals for a given macroarea) of F0–F3 (prevalence of 0.5%, 95% CI: 0.4–0.6%) were similar across the four macroareas (Table 1). While prevalence values in the Central and South regions for F4 stage disease (Fig. 2B) were approximately half of F0–F3 individuals (Fig. 2A), prevalence values for F4 individuals in the North region (Fig. 2B) were estimated to be about five-fold less than F0–F3 individuals (Fig. 2A).

**Table 1:** Estimates of the absolute number and % of viraemic HCV individuals in Italy according to fibrosis stage and macro area

Macroarea/fibrosis stage	Absolute number and 95% CI	Prevalence (%) and 95% CI (%)	Proportion (%)
Total	409,184 (325,007–494,591)	0.68 (0.54–0.82)	
Total F0–F3	300,171 (239,306–365,317)	0.50 (0.4–0.6)	100
Total F4	109,012 (84,886–135,012)	0.18 (0.14–0.22)	100
Macro regions			
North			
F0–F3	126,376 (100,711–153,938)	0.45 (0.36–0.55)	42.1
F4	27,999 (20,654–35,542)	0.10 (0.07–0.13)	25.7
Central			
F0–F3	72,453 (57,742–88,048)	0.61 (0.48–0.74)	24.1
F4	34,637 (27,160–42,360)	0.29 (0.23–0.35)	31.8
South			
F0–F3	69,540 (55,450–84,629)	0.50 (0.4–0.61)	23.2
F4	33,799 (26,606–41,515)	0.24 (0.19–0.3)	31.0
Isles			
F0–F3	31,802 (25,304–38,522)	0.49 (0.39–0.59)	10.6
F4	12,578 (9,809–15,614)	0.19 (0.15–0.24)	11.5

Fibrosis stages: F0–F3 = asymptomatic; undiagnosed/unlinked to care and F4 = symptomatic; potentially linked to care and cure

*CI* confidence intervals

### Estimation of the proportion of individuals across Italian regions

Despite similar prevalence values observed across the four macroareas, the 300,171 estimated individuals with F0–F3 fibrosis stage (potentially undiagnosed) were distributed across regions as follows: 42.1% in the North, followed by 24.1% in the Central to lower proportions observed in the South (23.2%), and the Isles (10.6%) (Table 1). Independent of similar prevalence estimates, the distribution of these individuals revealed the highest absolute numbers in the Lombardia region followed by Lazio, Campania, Veneto, Sicilia, Piemonte, Puglia, Toscana and Emilia Romagna (range: 19,374–41,543) (Table 2). The remaining regions were estimated to have lower numbers, ranging from 658 in Valle d’Aosta to more than 10,000 in Calabria and Marche.

**Table 2:** Estimates of the absolute number and % of viraemic HCV individuals in different Italian regions according to fibrosis stage

Region	Fibrosis stage	Absolute number			Prevalence (%)		
		Number	Lower 95% CI	Upper 95% CI	Mean	Lower 95% CI	Upper 95% CI
Abruzzo	F0–F3	7935	6327	9678	0.61	0.49	0.74
	F4	4295	3367	5223	0.33	0.26	0.4
Basilicata	F0–F3	3668	2918	4449	0.65	0.52	0.79
	F4	2022	1591	2466	0.36	0.28	0.44
Calabria	F0–F3	10,506	8312	12,854	0.53	0.42	0.65
	F4	5614	4424	6826	0.29	0.22	0.35
Campania	F0–F3	25,656	20,523	31,339	0.45	0.36	0.55
	F4	11,088	8493	13,784	0.19	0.15	0.24
Emilia-Romagna	F0–F3	19,374	15,478	23,569	0.43	0.34	0.52
	F4	3879	2808	5009	0.09	0.06	0.11
Friuli-Venezia Giulia	F0–F3	6149	4910	7448	0.5	0.4	0.6
	F4	1597	1209	2009	0.13	0.1	0.16
Lazio	F0–F3	35,804	28,516	43,440	0.62	0.5	0.76
	F4	17,534	13,712	21,359	0.31	0.24	0.37
Liguria	F0–F3	6542	5198	7946	0.42	0.33	0.51
	F4	1076	794	1374	0.07	0.05	0.09
Lombardia	F0–F3	41,543	33,179	50,408	0.41	0.33	0.49
	F4	6894	4839	9053	0.07	0.05	0.09
Marche	F0–F3	10,618	8434	12,970	0.68	0.54	0.83
	F4	5557	4399	6768	0.36	0.28	0.43
Molise	F0–F3	1688	1351	2051	0.55	0.44	0.66
	F4	851	658	1036	0.27	0.21	0.33
Piemonte	F0–F3	22,744	18,112	27,746	0.52	0.41	0.63
	F4	6535	4969	8175	0.15	0.11	0.19
Puglia	F0–F3	20,088	15,988	24,403	0.5	0.4	0.61
	F4	9930	7779	12,245	0.25	0.2	0.31
Sardegna	F0–F3	8614	6862	10,434	0.53	0.42	0.64
	F4	3693	2855	4562	0.23	0.18	0.28
Sicilia	F0–F3	23,188	18,480	28,138	0.47	0.38	0.57
	F4	8884	6932	11,032	0.18	0.14	0.23
Toscana	F0–F3	19,598	15,652	23,878	0.52	0.41	0.63
	F4	8173	6321	10,108	0.22	0.17	0.27
Trentino Alto Adige/Südtirol	F0–F3	5123	4108	6205	0.47	0.38	0.57
	F4	1352	1038	1679	0.12	0.1	0.15
Umbria	F0–F3	6433	5161	7814	0.72	0.58	0.88
	F4	3374	2654	4109	0.38	0.3	0.46
Valle d'Aosta/Vallée d'Aoste	F0–F3	658	531	797	0.52	0.42	0.64
	F4	183	137	229	0.15	0.11	0.18
Veneto	F0–F3	24,243	19,288	29,506	0.48	0.39	0.59
	F4	6483	4860	8171	0.13	0.1	0.16

Fibrosis stages: F0–F3 = asymptomatic; undiagnosed/unlinked to care and F4 = symptomatic; potentially linked to care and cure

*CI* confidence intervals

The distribution of individuals with stage F4 disease (109,012 estimated individuals) was highest in the Central and Southern regions (31.8% and 31.0%, respectively), followed by the Northern (25.7%) and the Isles (11.5%) regions (Table 1). Regions reporting the highest estimated numbers of individuals included Lazio (17,534) and Campania (11,088) (Table 2).

### Prevalence by high-risk transmission route

Although marked differences in prevalence estimates across the five high risk groups were observed (Table 3), with the highest seen in the PWID and tattoo groups, little variation in overall prevalence estimates was seen across the four macroareas for each infection route (Table 4). Despite similar prevalence estimates seen by macroarea in undiagnosed individuals (F0–F3 fibrosis stage; range: 0.23–0.31%), the estimated absolute number of PWIDs with F0–F3 (total of 151,296 individuals) was approximately two-fold higher in the Northern (64,922) than Central and Southern (36,674 and 34,432, respectively) regions, and approximately four-fold higher than the Isles region (15,268) (Table 4). Specific estimated numbers for each region by transmission route are shown in Additional file 1: Table S1.

**Table 3:** Estimates of the absolute number and % of viraemic HCV individuals in Italy according to fibrosis stage and route of transmission

Macroarea/fibrosis stage	Absolute number and 95% CI	Prevalence (%) and 95% CI (%)	Proportion (%)
Total	409,184 (325,007–494,591)	0.68 (0.54–0.82)	
Total F0–F3	300,171 (239,306–365,317)	0.50 (0.4–0.6)	100
Total F4	109,012 (84,886–135,012)	0.18 (0.14–0.22)	100
Transmission route			
GS + transfusion			
F0–F3	15,412 (12,155–18,986)	0.02 (0.02–0.03)	5.1
F4	44,595 (35,032–54,287)	0.07 (0.06–0.09)	40.9
PWID			
F0–F3	151,296 (120,700–184,164)	0.25 (0.2–0.3)	50.4
F4	47,514 (36,507–59,336)	0.08 (0.06–0.1)	43.6
Sex			
F0–F3	42,591 (33,928–51,625)	0.07 (0.06–0.09)	14.2
F4	3,249 (2393–4093)	0.01 (0–0.01)	3.0
Tattoo			
F0–F3	89,236 (71,153–108,577)	0.15 (0.12–0.18)	29.7
F4	12,414 (9177–15,912)	0.02 (0.02–0.03)	11.4
Vertical			
F0–F3	1,637 (1306–2008)	< 0.01 (< 0.01– < 0.01)	0.55
F4	1,241 (958–1523)	< 0.01 (< 0.01– < 0.01)	1.14

Fibrosis stages: F0–F3 = asymptomatic; undiagnosed/unlinked to care and F4 = symptomatic; potentially linked to care and cure

*CI* confidence intervals, *GS* glass syringe, *HCV* hepatitis C virus, *PWID* people who inject drugs

**Table 4:** Estimates of the absolute number and % of viraemic HCV individuals in Italy according to fibrosis stage, route of transmission and macro area

Macroarea	Transmission route	Fibrosis stage	Absolute number			Prevalence (%)		
			Number	Lower 95% CI	Upper 95% CI	Mean	Lower 95% CI	Upper 95% CI
North	GS + transfusion	F0–F3	3318	2591	4118	0.01	0.01	0.01
		F4	6817	5211	8384	0.02	0.02	0.03
	PWID	F0–F3	64,922	51,853	79,217	0.23	0.18	0.28
		F4	15,399	11,393	19,517	0.05	0.04	0.07
	Sex	F0–F3	18,722	14,870	22,705	0.07	0.05	0.08
		F4	1156	816	1482	< 0.01	< 0.01	0.01
	Tattoo	F0–F3	38,761	30,970	47,218	0.14	0.11	0.17
		F4	4260	3026	5614	0.02	0.01	0.02
	Vertical	F0–F3	652	508	827	< 0.01	< 0.01	< 0.01
		F4	367	271	474	< 0.01	< 0.01	< 0.01
Central	GS + transfusion	F0–F3	4713	3681	5780	0.04	0.03	0.05
		F4	14,882	11,763	18,069	0.12	0.1	0.15
	PWID	F0–F3	36,674	29,263	44,609	0.31	0.24	0.37
		F4	14,781	11,568	18,194	0.12	0.1	0.15
	Sex	F0–F3	9796	7819	11,863	0.08	0.07	0.1
		F4	922	690	1156	0.01	0.01	0.01
	Tattoo	F0–F3	20,894	16,638	25,354	0.17	0.14	0.21
		F4	3687	2793	4645	0.03	0.02	0.04
	Vertical	F0–F3	376	297	462	< 0.01	< 0.01	< 0.01
		F4	365	291	449	< 0.01	< 0.01	< 0.01
South	GS + transfusion	F0–F3	5423	4242	6733	0.04	0.03	0.05
		F4	17,035	13,367	20,767	0.12	0.1	0.15
	PWID	F0–F3	34,432	27,465	41,917	0.25	0.2	0.3
		F4	12,548	9707	15,594	0.09	0.07	0.11
	Sex	F0–F3	9444	7523	11,516	0.07	0.05	0.08
		F4	799	589	1003	0.01	< 0.01	0.01
	Tattoo	F0–F3	19,834	15,787	24,109	0.14	0.11	0.17
		F4	3064	2281	3925	0.02	0.02	0.03
	Vertical	F0–F3	407	316	512	< 0.01	< 0.01	< 0.01
		F4	354	281	439	< 0.01	< 0.01	< 0.01
Isles	GS + transfusion	F0–F3	1958	1525	2404	0.03	0.02	0.04
		F4	5861	4626	7192	0.09	0.07	0.11
	PWID	F0–F3	15,268	12,147	18,505	0.23	0.19	0.28
		F4	4787	3722	5964	0.07	0.06	0.09
	Sex	F0–F3	4628	3681	5632	0.07	0.06	0.09
		F4	372	274	484	0.01	< 0.01	0.01
	Tattoo	F0–F3	9748	7782	11,851	0.15	0.12	0.18
		F4	1403	1037	1843	0.02	0.02	0.03
	Vertical	F0–F3	201	155	253	< 0.01	< 0.01	< 0.01
		F4	155	111	202	< 0.01	< 0.01	< 0.01

Fibrosis stages: F0–F3 = asymptomatic; undiagnosed/unlinked to care and F4 = symptomatic; potentially linked to care and cure

*CI* confidence intervals, *GS* glass syringe, *HCV* hepatitis C virus, *PWID* people who inject drugs

The estimated number of patients with stage F4 disease in the PWID (total of 47,514 individuals) was highest in the Northern (15,399), followed by the Central (14,781), and the Southern (12,548), regions, and lower in the Isles region (4787) (Table 4).

### Prevalence by age range

**Fig. 3:**
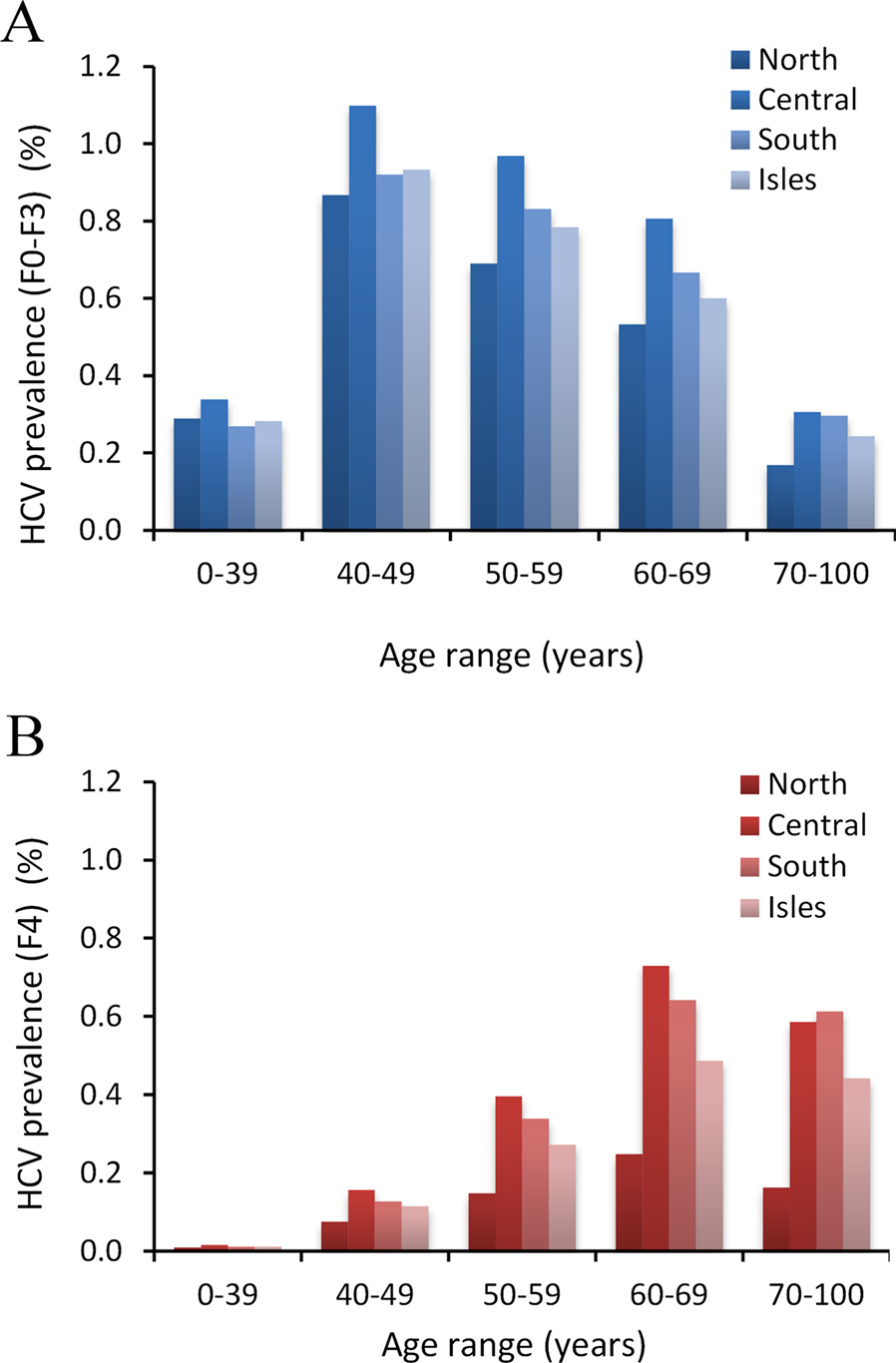
Estimated prevalence of HCV in different macroareas of Italy by age groups. **A** The prevalence of potentially undiagnosed infected individuals (stage F0–F3) and (**B**) infected individuals with stage F4 liver fibrosis (potentially diagnosed). Prevalence estimates are presented as percentage for the four macroareas. F0–F3 = asymptomatic; undiagnosed/unlinked to care; F4 = symptomatic; potentially linked to care and cure; HCV = hepatitis C virus

Prevalence estimates by age range are shown for stage F0–F3 and F4 disease in Fig. 3A, B, respectively. The highest prevalence estimates for individuals with stage F0–F3 were seen in the 40–49 years’ age groups for undiagnosed individuals, decreasing with increasing age, with similar profile maintained for each macroarea (Fig. 3A). In contrast, for individuals with F4 stage disease, the highest prevalence was focused in individuals 60 years and older (Fig. 3B).

### Sensitivity analysis

**Fig. 4:**
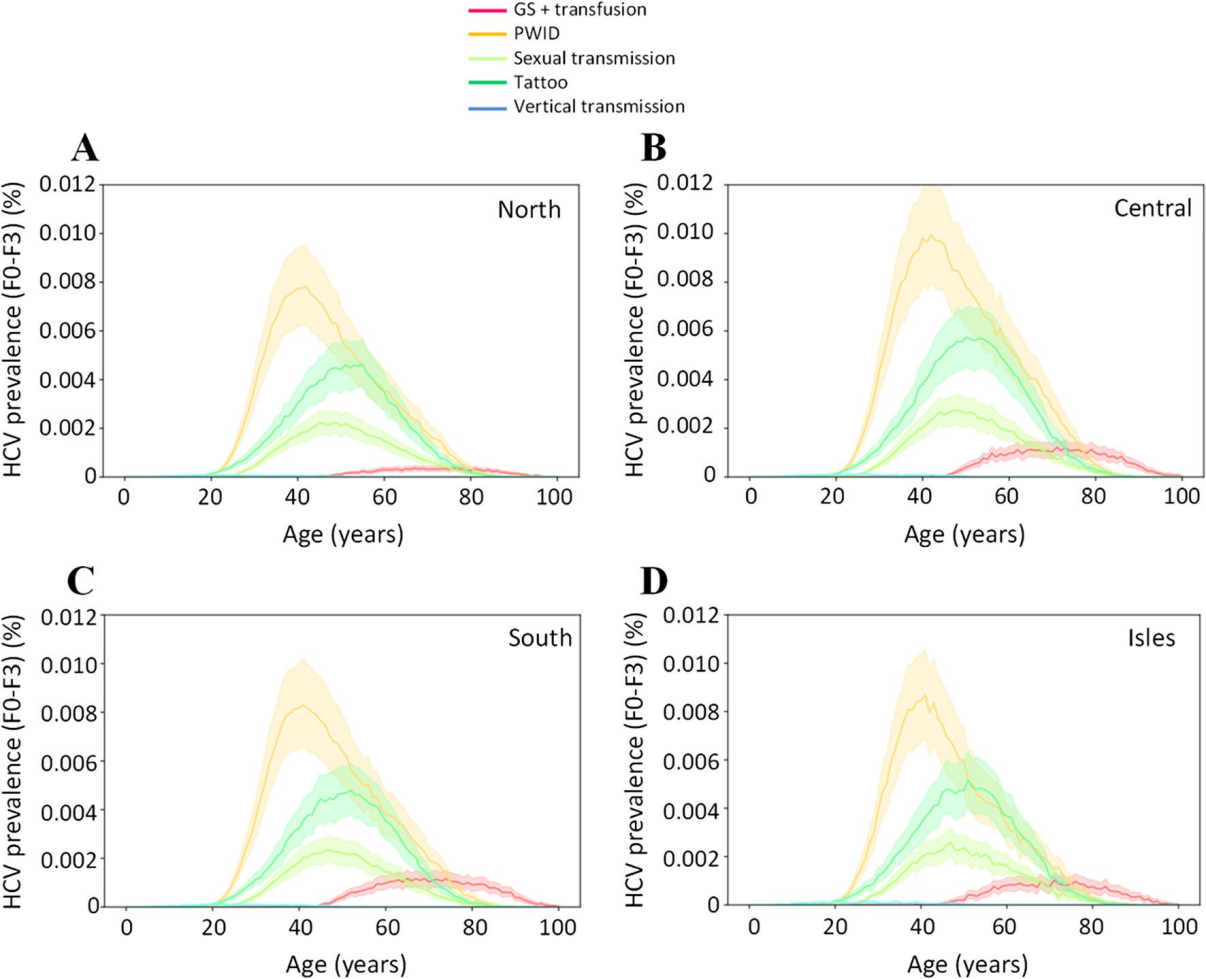
Probability distribution for the five high risk groups by age and fibrosis stage for the four macroareas (North, Central, South and Isles; **A**–**D**) in individuals with HCV with F0–F3 liver fibrosis stage. Solid lines represent the mean values. The shadow filled curves represent lower and upper 95% confidence intervals. F0–F3 = asymptomatic; undiagnosed/unlinked to care; GS = glass syringe; HCV = hepatitis C virus; PWID = people who inject drugs

**Fig. 5:**
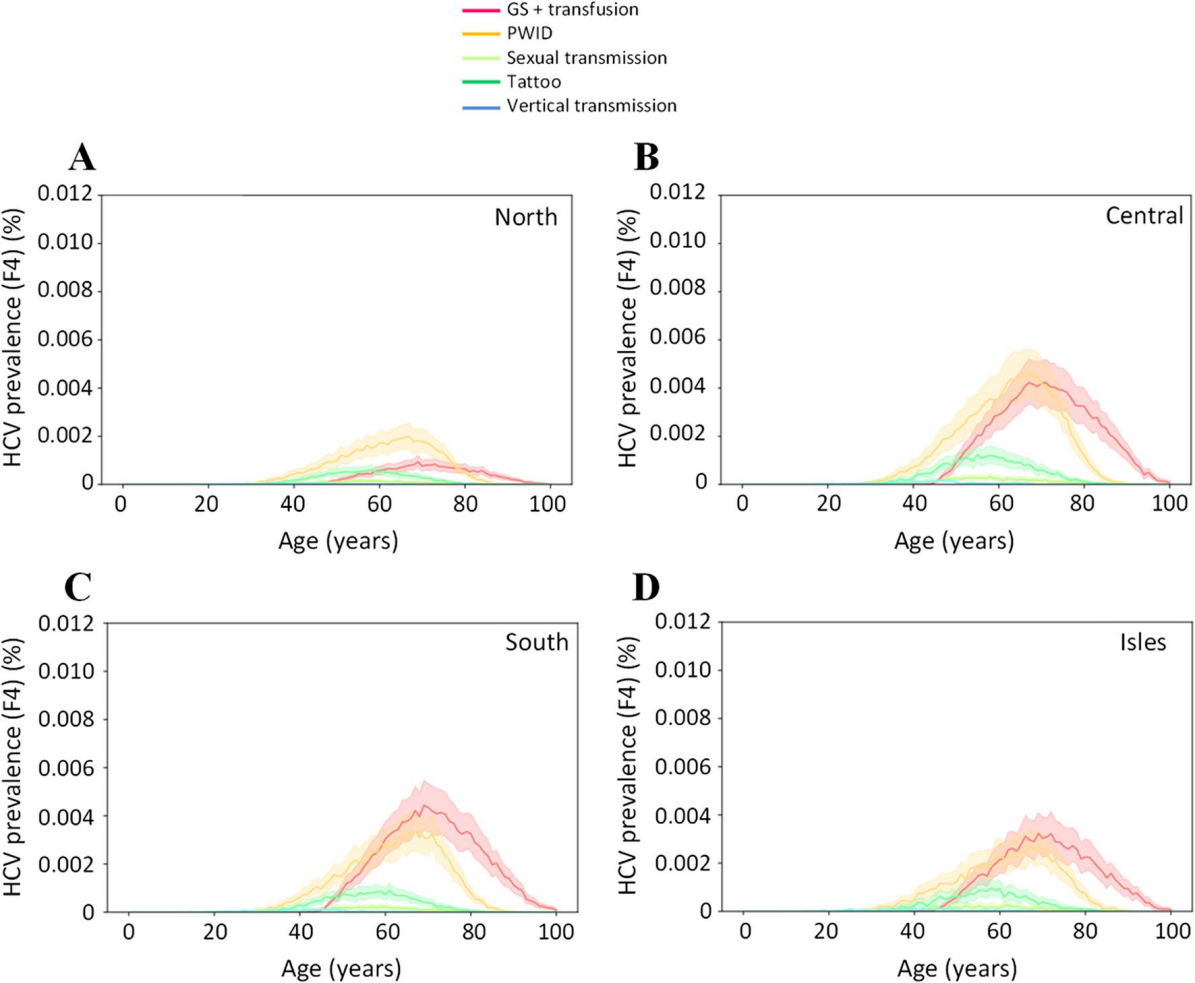
Probability distribution for the five high risk groups by age and fibrosis stage for the four macroareas (North, Central, South and Isles; **A**–**D**) in individuals with HCV with F4 liver fibrosis stage. Solid lines represent the mean values. The shadow filled curves represent lower and upper 95% confidence intervals. F4 = symptomatic; potentially linked to care and cure; GS = glass syringe; HCV = hepatitis C virus; PWID = people who inject drugs

The sensitivity of our model by route of transmission for the different macroareas for F0–F3 and F4 individuals are shown in Figs. 4 and 5, respectively. In individuals with F0–F3 disease (Fig. 4), variation around the mean (upper and lower 95% CIs) was narrow for the lower age categories (20–40 years) and widens for the peak prevalence, to a greater extent in the PWID group and sexual transmission compared with other groups. This trend remained similar across the four macroareas (Fig. 4A–D).

In individuals with F4 disease (Fig. 5), the pattern of infection across the four macroareas was different. The overall prevalence estimates were lower, particularly in the North (Fig. 5A), compared with the other macroareas (Fig. 5B–D). For F4 prevalence, the variation around the reference case is approximately proportional to the value of the reference case itself. Overall variation around mean values was 10%-15%, indicating robustness of the model.

## Discussion

Findings from the present study revealed that an increasing trend in the undiagnosed population with HCV (F0–F3) from South (23.2%) to North (42.1%), independent of similar regional prevalence values. PWID and tattoo risk emerged as the main populations of undiagnosed individuals, with similar distribution observed across regions.

The estimated number (and distribution across Italy) of patients with F4 cirrhosis is particularly concerning. This was unexpected, considering their potential symptomatic disease and the high importance of viral eradication in these patients, who were prioritized for treatment since 2015, when DAAs became available. In fact, around 20% of patients with cirrhosis are also observed in the AIFA treatment DAA monitoring registry for the year 2019 [16]. These data could suggest the lack of an adequate linkage to care in diagnosed individuals or the first diagnosis of these patients in very severe stages of liver disease, which again emphasises the increased need for screening and immediate linkage to care of individuals with HCV in Italy.

Although a higher estimated HCV prevalence of undiagnosed individuals emerged from some specific regions in Central Italy (e.g. Umbria and Marche) and in the Basilicata region in the South, similar lower prevalence estimates of undiagnosed individuals were generally observed across the four macroareas. This suggests a decrease in the level of prevalence in HCV infection compared to the past in Italy where higher prevalence values and a gradient from North to South (3.9% in Veneto to 16.2% in Campania) of individuals with HCV in Italy mainly related to the nosocomial transmission of infection [8, 27]. However, considering the distribution of the number of undiagnosed individuals (N = 300,171), an increasing trend as absolute number from the South (23.2%) to North (42.1%) emerged. Why our data suggest a different gradient as absolute estimates of undiagnosed individuals, which is higher in the Northern region compared with other macroareas, may be explained by some epidemiologic and sociodemographic features. First, considering the cohort effect of HCV infection in Italy, infections that occurred in the first wave of infection have a higher probability of being cured by now. Therefore, the high number of patients with fibrosis stage F4/cirrhosis receiving treatment in Italy at the beginning have contributed potentially to the substantial decrease in the number of infected individuals who have had severe liver damage (mainly prevalent in the South). The second wave was mainly associated with key populations (i.e. PWID and tattoos), and the more recent infection that has also been reported in Northern Europe [28, 29].

Second, the number of F0–F3 infections could be higher in the North compared with other macroareas due to the internal migration of the Italian population who are actively working (aged 30–60 years) from the South and Islands to the Northern areas, where there is a higher rate of employment and work activity in the country [30]. Of note, these data do not emerge when only the overall prevalence estimated for each macroarea is considered. This can be explained by the fact that the prevalence values consider the whole population with the same age and population, which is also higher in number (the denominator) in Northern compared with Southern Italy. This yields a similar estimated prevalence, despite high absolute infection burden of asymptomatic individuals in the North compared with other macroareas.

While these findings underline the high HCV burden of asymptomatic individuals in the North, substantial heterogeneity exists across regions, necessitating individual elimination plans to be implemented at the regional level.

The recommended screening approved by law starting with birth in the years 1969–1989 is in accordance with the results of this study, although derived using different modelling approaches [31, 32].

It is also worth mentioning that our analysis in the present study was performed up to January 2020, a few months before the severe acute respiratory syndrome coronavirus 2 (SARS-CoV-2) virus pandemic [33]. To cope with the increase in number of COVID-19 patients to emergency departments, the re-organization of healthcare facilities across all Italian regions was necessary resulting in the postponing of medical services and procedures considered as ‘non-essential’ or ‘deferrable’. The potential impact of this deferral has been assessed in a separate analysis by Kondili et al. [34]. In this modelling approach, it was estimated that deferring DAA treatment for an additional 6 months would, at 5 years, increase the number of HCV patients dying of a liver-related condition in Italy to more than 500 patients, deaths avoidable by a not deferred test and treatment approach. A further analysis also revealed that a 1-year delay in hepatitis diagnosis and treatment could result in an additional 44,800 liver cancers and 72,300 deaths from HCV globally by 2030 [35]. Regions such as Lombardy may be particularly susceptible to any deferral of DAA treatment [36], and strategies aimed to increase diagnosis and treatment are warranted.

### Limitations

This study has some potential limitations that need to be highlighted. The impact of less frequent routes of transmission (e.g. surgical interventions, colonoscopy, dental intervention/surgery, cosmetics) was not considered, potentially underestimating the number of both diagnosed and undiagnosed individuals.

We acknowledge that variation in prevalence rates was estimated considering baseline rates derived from studies that were not always designed as prevalence studies in the general population and for populations with different risk factors such as sexual transmission, which for this reason could have been underestimated. To partially overcome these limitations, the prevalence retrieved from these articles was not used as a uniform probability throughout all years and ages, but a shape (probability density function modelling) was derived from different sources to more realistically model the prevalence over different years. However, considering the high rate of treatment in Italy in the past 5 years and considering DAA treatment as a preventative measure, the rate of reinfection and new infection would be expected to be low, therefore reducing the extent of any potential underestimation.

We considered non-liver related mortality (i.e. natural mortality due to other comorbid diseases) based on ISTAT (Italian mortality registry) data that are usually used in modelling analysis of HCV natural history. This could have overestimated the alive population with HCV infection estimated. The non-liver related mortality population could be higher than those infected compared to the general population due to extrahepatic manifestations of HCV infection, placing them at higher risk of death earlier in life [37]. However, this probability has not been modelled due to the lack of numerical data.

A high number of unregistered immigrants (potentially asymptomatic and undiagnosed for HCV) travelling to Italy in recent years [38, 39], and particularly in the Northern regions [40] where the highest number of individuals with HCV was observed in our model. This aspect needs to be carefully considered during the interpretation of our estimates. However, based on expert opinion and data derived from other studies, a higher HCV prevalence is not observed in this immigrant population (considering their countries of birth) compared to the Italian population (resident in Italy that are included in general population estimates) [41]. In separate analyses, we are working to address not only the higher prevalence rate, but also the impact that this brings to society. In this study, the main routes of infections have been considered separately in the evaluation of the number of untreated individuals in Italy and we have not focussed on specific settings (e.g. immigrant or prison population) mainly due to the lack of reliable National data. Further studies should explore the impact of the immigrant population on HCV prevalence separately.

With regard to the prison population, most prisoners have a history of high-risk sexual behavior, injection drug use and tattooing and it appears that the risk of acquisition of HCV infection is linked to these behaviours which are considered in this modelling. For the aim of this study, in the evaluation of the number of untreated individuals in Italy, the main routes of infection were considered separately by risk factor and not specific settings such as homeless, migrants etc., mainly due to the lack of reliable National data. The prison per se was not considered a route of infection, but PWID is recognised as a main route of infection in prison [42–44] rather than the prisoning state itself, and this could result in potential underestimation bias and this could result in potential underestimation bias.

Transition probabilities may vary for different populations that depend on host response and underlying comorbidities [37]. We have aimed to minimize this variation by considering this uncertainty in the sensitivity analysis.

In this model, we assumed that all F0–F3 infected individuals estimated in the model that were not yet treated as undiagnosed. In the real-life scenario, not all F0–F3 are undiagnosed and therefore a portion of these individuals could be diagnosed and not yet linked to care. Regardless, these data do not influence the infection burden, because although these individuals could be diagnosed, they are estimated as not treated.

## Conclusion

By January 2020, the number of individuals with HCV in Italy was estimated at 409,184 (prevalence of 0.68%; 95% CI: 0.54–0.82%), of which 300,171 (0.50%; 95% CI: 0.4–0.6%) were estimated as undiagnosed due to their asymptomatic disease (F0–F3). The target for new diagnosis should focus and screening of PWID, tattoo, and sexual transmission, in younger people (predominantly aged 40–60 years) should be implemented.

An increasing trend in the percentage of the undiagnosed population with HCV (F0–F3) from South (23.2%) to North (42.1%) has emerged, independent of similar regional prevalence values. PWID and those who received tattoos in the past represent the main populations of undiagnosed individuals, with similar distributions across regions. This targeted modelling approach, which addresses the specific profile of undiagnosed individuals, is helpful in planning effective elimination strategies by region in Italy and could be a useful methodology for other countries in implementing their elimination plans.

## Supplementary Information

**Additional file 1: Table S1. **Estimates of % of viraemic HCV individuals in different Italian regions according to fibrosis stage and high-risk groups.

## Data Availability

All datasets presented in this study are included in the article/Additional file. Additional data can be made available upon request from AD and RM.
