# Supplementary material for: A mathematical model by route of transmission and fibrosis progression to estimate undiagnosed individuals with HCV in different Italian regions
Source: BMC Infect Dis. 2022 Jan 17;22:58. doi: 10.1186/s12879-022-07042-w (PMC8761836; doi:10.1186/s12879-022-07042-w)
Supplement: Supplementary file 1 — Additional file 1: Table S1. Estimates of % of viraemic HCV individuals in different Italian regions according to fibrosis stage and high-risk groups. [file 12879_2022_7042_MOESM1_ESM.docx]

Supplementary Material 1. Estimates of % of viraemic HCV individuals in different Italian regions according to fibrosis stage and high-risk groups.

|  |  |  | Absolute number | | | Prevalence (%) | | |
| --- | --- | --- | --- | --- | --- | --- | --- | --- |
| Region | Transmission route | Fibrosis stage | Number | Lower 95% CI | Upper 95% CI | Mean | Lower 95% CI | Upper 95% CI |
| Abruzzo | GS | F0-F3 | 452 | 349 | 570 | 0.03 | 0.03 | 0.04 |
|  |  | F4 | 1767 | 1387 | 2159 | 0.14 | 0.11 | 0.17 |
|  | PWID | F0-F3 | 4071 | 3253 | 4950 | 0.31 | 0.25 | 0.38 |
|  |  | F4 | 1712 | 1325 | 2104 | 0.13 | 0.1 | 0.16 |
|  | Sex | F0-F3 | 1011 | 799 | 1236 | 0.08 | 0.06 | 0.09 |
|  |  | F4 | 94 | 64 | 126 | 0.01 | <0.01 | 0.01 |
|  | Tattoo | F0-F3 | 2148 | 1703 | 2637 | 0.16 | 0.13 | 0.2 |
|  |  | F4 | 391 | 289 | 486 | 0.03 | 0.02 | 0.04 |
|  | transfusion | F0-F3 | 218 | 163 | 277 | 0.02 | 0.01 | 0.02 |
|  |  | F4 | 299 | 221 | 382 | 0.02 | 0.02 | 0.03 |
|  | vertical | F0-F3 | 36 | 27 | 46 | <0.01 | <0.01 | <0.01 |
|  |  | F4 | 32 | 25 | 42 | <0.01 | <0.01 | <0.01 |
| Basilicata | GS | F0-F3 | 254 | 196 | 326 | 0.05 | 0.03 | 0.06 |
|  |  | F4 | 1007 | 782 | 1234 | 0.18 | 0.14 | 0.22 |
|  | PWID | F0-F3 | 1616 | 1273 | 1978 | 0.29 | 0.23 | 0.35 |
|  |  | F4 | 601 | 474 | 735 | 0.11 | 0.08 | 0.13 |
|  | Sex | F0-F3 | 507 | 401 | 623 | 0.09 | 0.07 | 0.11 |
|  |  | F4 | 48 | 32 | 66 | 0.01 | 0.01 | 0.01 |
|  | Tattoo | F0-F3 | 1147 | 916 | 1378 | 0.2 | 0.16 | 0.24 |
|  |  | F4 | 190 | 137 | 258 | 0.03 | 0.02 | 0.05 |
|  | transfusion | F0-F3 | 121 | 94 | 158 | 0.02 | 0.02 | 0.03 |
|  |  | F4 | 166 | 122 | 214 | 0.03 | 0.02 | 0.04 |
|  | vertical | F0-F3 | 24 | 20 | 29 | <0.01 | <0.01 | 0.01 |
|  |  | F4 | 10 | 6 | 15 | <0.01 | <0.01 | <0.01 |
| Calabria | GS | F0-F3 | 611 | 467 | 764 | 0.03 | 0.02 | 0.04 |
|  |  | F4 | 2461 | 1953 | 2997 | 0.13 | 0.1 | 0.15 |
|  | PWID | F0-F3 | 5100 | 4029 | 6220 | 0.26 | 0.2 | 0.32 |
|  |  | F4 | 1987 | 1563 | 2433 | 0.1 | 0.08 | 0.12 |
|  | Sex | F0-F3 | 1416 | 1111 | 1736 | 0.07 | 0.06 | 0.09 |
|  |  | F4 | 144 | 108 | 190 | 0.01 | 0.01 | 0.01 |
|  | Tattoo | F0-F3 | 3032 | 2402 | 3714 | 0.15 | 0.12 | 0.19 |
|  |  | F4 | 515 | 388 | 667 | 0.03 | 0.02 | 0.03 |
|  | transfusion | F0-F3 | 281 | 215 | 358 | 0.01 | 0.01 | 0.02 |
|  |  | F4 | 450 | 343 | 563 | 0.02 | 0.02 | 0.03 |
|  | vertical | F0-F3 | 67 | 47 | 86 | <0.01 | <0.01 | <0.01 |
|  |  | F4 | 57 | 45 | 74 | <0.01 | <0.01 | <0.01 |
| Campania | GS | F0-F3 | 1234 | 954 | 1553 | 0.02 | 0.02 | 0.03 |
|  |  | F4 | 4639 | 3599 | 5698 | 0.08 | 0.06 | 0.1 |
|  | PWID | F0-F3 | 12727 | 10204 | 15529 | 0.22 | 0.18 | 0.27 |
|  |  | F4 | 4227 | 3162 | 5295 | 0.07 | 0.06 | 0.09 |
|  | Sex | F0-F3 | 3591 | 2856 | 4364 | 0.06 | 0.05 | 0.08 |
|  |  | F4 | 275 | 200 | 365 | <0.01 | <0.01 | 0.01 |
|  | Tattoo | F0-F3 | 7351 | 5869 | 8959 | 0.13 | 0.1 | 0.16 |
|  |  | F4 | 999 | 711 | 1292 | 0.02 | 0.01 | 0.02 |
|  | transfusion | F0-F3 | 600 | 465 | 751 | 0.01 | 0.01 | 0.01 |
|  |  | F4 | 810 | 616 | 1019 | 0.01 | 0.01 | 0.02 |
|  | vertical | F0-F3 | 153 | 111 | 207 | <0.01 | <0.01 | <0.01 |
|  |  | F4 | 139 | 101 | 183 | <0.01 | <0.01 | <0.01 |
| Emilia-Romagna | GS | F0-F3 | 374 | 281 | 473 | 0.01 | 0.01 | 0.01 |
|  |  | F4 | 855 | 630 | 1091 | 0.02 | 0.01 | 0.02 |
|  | PWID | F0-F3 | 9951 | 7957 | 12119 | 0.22 | 0.18 | 0.27 |
|  |  | F4 | 2045 | 1470 | 2648 | 0.05 | 0.03 | 0.06 |
|  | Sex | F0-F3 | 2858 | 2247 | 3495 | 0.06 | 0.05 | 0.08 |
|  |  | F4 | 164 | 112 | 217 | <0.01 | <0.01 | <0.01 |
|  | Tattoo | F0-F3 | 5905 | 4695 | 7185 | 0.13 | 0.1 | 0.16 |
|  |  | F4 | 589 | 407 | 795 | 0.01 | 0.01 | 0.02 |
|  | transfusion | F0-F3 | 189 | 144 | 252 | <0.01 | <0.01 | <0.01 |
|  |  | F4 | 169 | 121 | 222 | <0.01 | <0.01 | <0.01 |
|  | vertical | F0-F3 | 97 | 68 | 128 | <0.01 | <0.01 | <0.01 |
|  |  | F4 | 57 | 37 | 78 | <0.01 | <0.01 | <0.01 |
| Friuli-Venezia Giulia | GS | F0-F3 | 112 | 82 | 150 | 0.01 | 0.01 | 0.01 |
|  |  | F4 | 337 | 244 | 436 | 0.03 | 0.02 | 0.04 |
|  | PWID | F0-F3 | 3068 | 2445 | 3714 | 0.25 | 0.2 | 0.3 |
|  |  | F4 | 855 | 649 | 1084 | 0.07 | 0.05 | 0.09 |
|  | Sex | F0-F3 | 919 | 730 | 1122 | 0.07 | 0.06 | 0.09 |
|  |  | F4 | 55 | 32 | 81 | <0.01 | <0.01 | 0.01 |
|  | Tattoo | F0-F3 | 1946 | 1545 | 2369 | 0.16 | 0.13 | 0.19 |
|  |  | F4 | 262 | 189 | 343 | 0.02 | 0.02 | 0.03 |
|  | transfusion | F0-F3 | 62 | 43 | 86 | <0.01 | <0.01 | 0.01 |
|  |  | F4 | 70 | 50 | 98 | 0.01 | <0.01 | 0.01 |
|  | vertical | F0-F3 | 42 | 34 | 53 | <0.01 | <0.01 | <0.01 |
|  |  | F4 | 17 | 11 | 23 | <0.01 | <0.01 | <0.01 |
| Lazio | GS | F0-F3 | 1648 | 1269 | 2042 | 0.03 | 0.02 | 0.04 |
|  |  | F4 | 6700 | 5279 | 8153 | 0.12 | 0.09 | 0.14 |
|  | PWID | F0-F3 | 17515 | 13950 | 21226 | 0.31 | 0.24 | 0.37 |
|  |  | F4 | 6857 | 5354 | 8435 | 0.12 | 0.09 | 0.15 |
|  | Sex | F0-F3 | 4969 | 3962 | 6028 | 0.09 | 0.07 | 0.1 |
|  |  | F4 | 493 | 362 | 622 | 0.01 | 0.01 | 0.01 |
|  | Tattoo | F0-F3 | 10707 | 8507 | 12997 | 0.19 | 0.15 | 0.23 |
|  |  | F4 | 1973 | 1504 | 2485 | 0.03 | 0.03 | 0.04 |
|  | transfusion | F0-F3 | 757 | 575 | 943 | 0.01 | 0.01 | 0.02 |
|  |  | F4 | 1302 | 1036 | 1578 | 0.02 | 0.02 | 0.03 |
|  | vertical | F0-F3 | 209 | 159 | 263 | <0.01 | <0.01 | <0.01 |
|  |  | F4 | 208 | 156 | 264 | <0.01 | <0.01 | <0.01 |
| Liguria | GS | F0-F3 | 108 | 74 | 146 | 0.01 | <0.01 | 0.01 |
|  |  | F4 | 251 | 189 | 328 | 0.02 | 0.01 | 0.02 |
|  | PWID | F0-F3 | 3147 | 2505 | 3838 | 0.2 | 0.16 | 0.25 |
|  |  | F4 | 503 | 363 | 651 | 0.03 | 0.02 | 0.04 |
|  | Sex | F0-F3 | 1068 | 857 | 1296 | 0.07 | 0.06 | 0.08 |
|  |  | F4 | 59 | 40 | 79 | <0.01 | <0.01 | 0.01 |
|  | Tattoo | F0-F3 | 2123 | 1671 | 2598 | 0.14 | 0.11 | 0.17 |
|  |  | F4 | 201 | 144 | 273 | 0.01 | 0.01 | 0.02 |
|  | transfusion | F0-F3 | 59 | 41 | 80 | <0.01 | <0.01 | 0.01 |
|  |  | F4 | 48 | 33 | 67 | <0.01 | <0.01 | <0.01 |
|  | vertical | F0-F3 | 37 | 28 | 48 | <0.01 | <0.01 | <0.01 |
|  |  | F4 | 14 | 9 | 20 | <0.01 | <0.01 | <0.01 |
| Lombardia | GS | F0-F3 | 661 | 497 | 826 | 0.01 | <0.01 | 0.01 |
|  |  | F4 | 1385 | 991 | 1797 | 0.01 | 0.01 | 0.02 |
|  | PWID | F0-F3 | 21110 | 16853 | 25680 | 0.21 | 0.17 | 0.25 |
|  |  | F4 | 3747 | 2651 | 4937 | 0.04 | 0.03 | 0.05 |
|  | Sex | F0-F3 | 6299 | 4986 | 7634 | 0.06 | 0.05 | 0.07 |
|  |  | F4 | 296 | 200 | 405 | <0.01 | <0.01 | <0.01 |
|  | Tattoo | F0-F3 | 12884 | 10287 | 15655 | 0.13 | 0.1 | 0.15 |
|  |  | F4 | 1099 | 733 | 1482 | 0.01 | 0.01 | 0.01 |
|  | transfusion | F0-F3 | 378 | 295 | 478 | <0.01 | <0.01 | <0.01 |
|  |  | F4 | 276 | 195 | 364 | <0.01 | <0.01 | <0.01 |
|  | vertical | F0-F3 | 211 | 155 | 280 | <0.01 | <0.01 | <0.01 |
|  |  | F4 | 91 | 57 | 131 | <0.01 | <0.01 | <0.01 |
| Marche | GS | F0-F3 | 518 | 405 | 638 | 0.03 | 0.03 | 0.04 |
|  |  | F4 | 1953 | 1566 | 2383 | 0.13 | 0.1 | 0.15 |
|  | PWID | F0-F3 | 5432 | 4311 | 6674 | 0.35 | 0.28 | 0.43 |
|  |  | F4 | 2472 | 1939 | 3024 | 0.16 | 0.12 | 0.19 |
|  | Sex | F0-F3 | 1383 | 1101 | 1675 | 0.09 | 0.07 | 0.11 |
|  |  | F4 | 143 | 105 | 184 | 0.01 | 0.01 | 0.01 |
|  | Tattoo | F0-F3 | 2997 | 2377 | 3649 | 0.19 | 0.15 | 0.23 |
|  |  | F4 | 558 | 424 | 704 | 0.04 | 0.03 | 0.05 |
|  | transfusion | F0-F3 | 242 | 184 | 302 | 0.02 | 0.01 | 0.02 |
|  |  | F4 | 370 | 288 | 447 | 0.02 | 0.02 | 0.03 |
|  | vertical | F0-F3 | 46 | 35 | 57 | <0.01 | <0.01 | <0.01 |
|  |  | F4 | 62 | 54 | 71 | <0.01 | <0.01 | <0.01 |
| Molise | GS | F0-F3 | 102 | 70 | 140 | 0.03 | 0.02 | 0.05 |
|  |  | F4 | 443 | 342 | 551 | 0.14 | 0.11 | 0.18 |
|  | PWID | F0-F3 | 723 | 572 | 887 | 0.23 | 0.18 | 0.29 |
|  |  | F4 | 198 | 146 | 256 | 0.06 | 0.05 | 0.08 |
|  | Sex | F0-F3 | 246 | 192 | 304 | 0.08 | 0.06 | 0.1 |
|  |  | F4 | 24 | 14 | 35 | 0.01 | <0.01 | 0.01 |
|  | Tattoo | F0-F3 | 556 | 441 | 670 | 0.18 | 0.14 | 0.22 |
|  |  | F4 | 90 | 64 | 123 | 0.03 | 0.02 | 0.04 |
|  | transfusion | F0-F3 | 55 | 39 | 75 | 0.02 | 0.01 | 0.02 |
|  |  | F4 | 92 | 69 | 121 | 0.03 | 0.02 | 0.04 |
|  | vertical | F0-F3 | 6 | 5 | 9 | <0.01 | <0.01 | <0.01 |
|  |  | F4 | 3 | 1 | 6 | <0.01 | <0.01 | <0.01 |
| Piemonte | GS | F0-F3 | 389 | 291 | 503 | 0.01 | 0.01 | 0.01 |
|  |  | F4 | 1181 | 893 | 1473 | 0.03 | 0.02 | 0.03 |
|  | PWID | F0-F3 | 12339 | 9800 | 15037 | 0.28 | 0.22 | 0.34 |
|  |  | F4 | 3943 | 2986 | 4908 | 0.09 | 0.07 | 0.11 |
|  | Sex | F0-F3 | 3133 | 2500 | 3811 | 0.07 | 0.06 | 0.09 |
|  |  | F4 | 246 | 178 | 326 | 0.01 | <0.01 | 0.01 |
|  | Tattoo | F0-F3 | 6586 | 5235 | 8032 | 0.15 | 0.12 | 0.18 |
|  |  | F4 | 878 | 639 | 1125 | 0.02 | 0.01 | 0.03 |
|  | transfusion | F0-F3 | 193 | 147 | 252 | <0.01 | <0.01 | 0.01 |
|  |  | F4 | 214 | 160 | 271 | <0.01 | <0.01 | 0.01 |
|  | vertical | F0-F3 | 104 | 72 | 147 | <0.01 | <0.01 | <0.01 |
|  |  | F4 | 74 | 48 | 101 | <0.01 | <0.01 | <0.01 |
| Puglia | GS | F0-F3 | 975 | 748 | 1229 | 0.02 | 0.02 | 0.03 |
|  |  | F4 | 4156 | 3272 | 5074 | 0.1 | 0.08 | 0.13 |
|  | PWID | F0-F3 | 10196 | 8093 | 12381 | 0.26 | 0.2 | 0.31 |
|  |  | F4 | 3823 | 2962 | 4768 | 0.1 | 0.07 | 0.12 |
|  | Sex | F0-F3 | 2674 | 2119 | 3293 | 0.07 | 0.05 | 0.08 |
|  |  | F4 | 214 | 152 | 284 | 0.01 | <0.01 | 0.01 |
|  | Tattoo | F0-F3 | 5601 | 4456 | 6804 | 0.14 | 0.11 | 0.17 |
|  |  | F4 | 879 | 655 | 1132 | 0.02 | 0.02 | 0.03 |
|  | transfusion | F0-F3 | 521 | 401 | 646 | 0.01 | 0.01 | 0.02 |
|  |  | F4 | 744 | 585 | 923 | 0.02 | 0.01 | 0.02 |
|  | vertical | F0-F3 | 121 | 91 | 154 | <0.01 | <0.01 | <0.01 |
|  |  | F4 | 113 | 82 | 149 | <0.01 | <0.01 | <0.01 |
| Sardegna | GS | F0-F3 | 344 | 264 | 440 | 0.02 | 0.02 | 0.03 |
|  |  | F4 | 1259 | 984 | 1551 | 0.08 | 0.06 | 0.1 |
|  | PWID | F0-F3 | 4351 | 3413 | 5311 | 0.27 | 0.21 | 0.33 |
|  |  | F4 | 1680 | 1296 | 2085 | 0.1 | 0.08 | 0.13 |
|  | Sex | F0-F3 | 1175 | 938 | 1432 | 0.07 | 0.06 | 0.09 |
|  |  | F4 | 93 | 65 | 130 | 0.01 | <0.01 | 0.01 |
|  | Tattoo | F0-F3 | 2496 | 1993 | 3032 | 0.15 | 0.12 | 0.19 |
|  |  | F4 | 386 | 281 | 512 | 0.02 | 0.02 | 0.03 |
|  | transfusion | F0-F3 | 197 | 156 | 240 | 0.01 | 0.01 | 0.01 |
|  |  | F4 | 245 | 187 | 315 | 0.02 | 0.01 | 0.02 |
|  | vertical | F0-F3 | 51 | 36 | 68 | <0.01 | <0.01 | <0.01 |
|  |  | F4 | 30 | 20 | 43 | <0.01 | <0.01 | <0.01 |
| Sicilia | GS | F0-F3 | 962 | 734 | 1189 | 0.02 | 0.02 | 0.02 |
|  |  | F4 | 3679 | 2887 | 4512 | 0.08 | 0.06 | 0.09 |
|  | PWID | F0-F3 | 10917 | 8696 | 13245 | 0.22 | 0.18 | 0.27 |
|  |  | F4 | 3107 | 2399 | 3886 | 0.06 | 0.05 | 0.08 |
|  | Sex | F0-F3 | 3453 | 2742 | 4205 | 0.07 | 0.06 | 0.09 |
|  |  | F4 | 279 | 202 | 367 | 0.01 | <0.01 | 0.01 |
|  | Tattoo | F0-F3 | 7251 | 5769 | 8838 | 0.15 | 0.12 | 0.18 |
|  |  | F4 | 1017 | 739 | 1328 | 0.02 | 0.02 | 0.03 |
|  | transfusion | F0-F3 | 455 | 347 | 574 | 0.01 | 0.01 | 0.01 |
|  |  | F4 | 678 | 527 | 843 | 0.01 | 0.01 | 0.02 |
|  | vertical | F0-F3 | 150 | 113 | 194 | <0.01 | <0.01 | <0.01 |
|  |  | F4 | 125 | 88 | 165 | <0.01 | <0.01 | <0.01 |
| Toscana | GS | F0-F3 | 756 | 587 | 935 | 0.02 | 0.02 | 0.02 |
|  |  | F4 | 2655 | 2057 | 3262 | 0.07 | 0.05 | 0.09 |
|  | PWID | F0-F3 | 10211 | 8147 | 12435 | 0.27 | 0.22 | 0.33 |
|  |  | F4 | 3836 | 2969 | 4722 | 0.1 | 0.08 | 0.13 |
|  | Sex | F0-F3 | 2646 | 2107 | 3228 | 0.07 | 0.06 | 0.09 |
|  |  | F4 | 205 | 144 | 277 | 0.01 | <0.01 | 0.01 |
|  | Tattoo | F0-F3 | 5494 | 4390 | 6682 | 0.15 | 0.12 | 0.18 |
|  |  | F4 | 858 | 627 | 1109 | 0.02 | 0.02 | 0.03 |
|  | transfusion | F0-F3 | 401 | 313 | 504 | 0.01 | 0.01 | 0.01 |
|  |  | F4 | 548 | 426 | 678 | 0.01 | 0.01 | 0.02 |
|  | vertical | F0-F3 | 90 | 63 | 119 | <0.01 | <0.01 | <0.01 |
|  |  | F4 | 71 | 51 | 98 | <0.01 | <0.01 | <0.01 |
| Trentino Alto Adige / Südtirol | GS | F0-F3 | 90 | 63 | 123 | 0.01 | 0.01 | 0.01 |
|  |  | F4 | 361 | 284 | 439 | 0.03 | 0.03 | 0.04 |
|  | PWID | F0-F3 | 2418 | 1918 | 2947 | 0.22 | 0.18 | 0.27 |
|  |  | F4 | 633 | 475 | 785 | 0.06 | 0.04 | 0.07 |
|  | Sex | F0-F3 | 814 | 652 | 998 | 0.07 | 0.06 | 0.09 |
|  |  | F4 | 57 | 36 | 80 | 0.01 | <0.01 | 0.01 |
|  | Tattoo | F0-F3 | 1713 | 1366 | 2070 | 0.16 | 0.13 | 0.19 |
|  |  | F4 | 215 | 149 | 290 | 0.02 | 0.01 | 0.03 |
|  | transfusion | F0-F3 | 55 | 39 | 73 | 0.01 | <0.01 | 0.01 |
|  |  | F4 | 65 | 47 | 88 | 0.01 | <0.01 | 0.01 |
|  | vertical | F0-F3 | 33 | 26 | 42 | <0.01 | <0.01 | <0.01 |
|  |  | F4 | 21 | 16 | 28 | <0.01 | <0.01 | <0.01 |
| Umbria | GS | F0-F3 | 263 | 200 | 327 | 0.03 | 0.02 | 0.04 |
|  |  | F4 | 1145 | 909 | 1397 | 0.13 | 0.1 | 0.16 |
|  | PWID | F0-F3 | 3516 | 2819 | 4261 | 0.4 | 0.32 | 0.48 |
|  |  | F4 | 1616 | 1239 | 1999 | 0.18 | 0.14 | 0.22 |
|  | Sex | F0-F3 | 798 | 634 | 962 | 0.09 | 0.07 | 0.11 |
|  |  | F4 | 81 | 59 | 107 | 0.01 | 0.01 | 0.01 |
|  | Tattoo | F0-F3 | 1695 | 1343 | 2078 | 0.19 | 0.15 | 0.23 |
|  |  | F4 | 298 | 223 | 381 | 0.03 | 0.03 | 0.04 |
|  | transfusion | F0-F3 | 129 | 94 | 163 | 0.01 | 0.01 | 0.02 |
|  |  | F4 | 209 | 160 | 259 | 0.02 | 0.02 | 0.03 |
|  | vertical | F0-F3 | 32 | 26 | 39 | <0.01 | <0.01 | <0.01 |
|  |  | F4 | 25 | 19 | 33 | <0.01 | <0.01 | <0.01 |
| Valle d'Aosta / Vallée d'Aoste | GS | F0-F3 | 9 | 6 | 14 | 0.01 | 0.01 | 0.01 |
|  |  | F4 | 27 | 17 | 39 | 0.02 | 0.01 | 0.03 |
|  | PWID | F0-F3 | 354 | 280 | 435 | 0.28 | 0.22 | 0.35 |
|  |  | F4 | 106 | 73 | 137 | 0.08 | 0.06 | 0.11 |
|  | Sex | F0-F3 | 100 | 78 | 125 | 0.08 | 0.06 | 0.1 |
|  |  | F4 | 8 | 5 | 12 | 0.01 | <0.01 | 0.01 |
|  | Tattoo | F0-F3 | 193 | 150 | 248 | 0.15 | 0.12 | 0.2 |
|  |  | F4 | 37 | 27 | 51 | 0.03 | 0.02 | 0.04 |
|  | transfusion | F0-F3 | 1 | 0 | 4 | <0.01 | <0.01 | <0.01 |
|  |  | F4 | 5 | 4 | 9 | <0.01 | <0.01 | 0.01 |
|  | vertical | F0-F3 | 1 | 0 | 3 | <0.01 | <0.01 | <0.01 |
| Veneto | GS | F0-F3 | 433 | 327 | 544 | 0.01 | 0.01 | 0.01 |
|  |  | F4 | 1305 | 1017 | 1618 | 0.03 | 0.02 | 0.03 |
|  | PWID | F0-F3 | 12536 | 9983 | 15288 | 0.25 | 0.2 | 0.31 |
|  |  | F4 | 3567 | 2680 | 4521 | 0.07 | 0.05 | 0.09 |
|  | Sex | F0-F3 | 3531 | 2802 | 4293 | 0.07 | 0.06 | 0.09 |
|  |  | F4 | 271 | 199 | 353 | 0.01 | <0.01 | 0.01 |
|  | Tattoo | F0-F3 | 7411 | 5903 | 9025 | 0.15 | 0.12 | 0.18 |
|  |  | F4 | 979 | 703 | 1259 | 0.02 | 0.01 | 0.03 |
|  | transfusion | F0-F3 | 207 | 153 | 269 | <0.01 | <0.01 | 0.01 |
|  |  | F4 | 267 | 204 | 339 | 0.01 | <0.01 | 0.01 |
|  | vertical | F0-F3 | 127 | 91 | 164 | <0.01 | <0.01 | <0.01 |
|  |  | F4 | 94 | 66 | 127 | <0.01 | <0.01 | <0.01 |

GS = glass syringe, PWID = people who inject drugs

Fibrosis stages: F0-F3 = asymptomatic; undiagnosed/unlinked to care and F4 = symptomatic; potentially linked to care and cure.
